# Supplementary material for: Modeling glioblastoma heterogeneity as a dynamic network of cell states
Source: Mol Syst Biol. 2021 Sep 16;17(9):e10105. doi: 10.15252/msb.202010105 (PMC8444284; doi:10.15252/msb.202010105)
Supplement: Supplementary file 6 — Source Data for Figure 5 [file MSB-17-e10105-s004.zip › Figure5A_sourcedata/GSEA_3017/hallmarks_stateD.GseaPreranked.1621934542601/HALLMARK_COMPLEMENT.html]

Details for gene set HALLMARK\_COMPLEMENT[GSEA]

|  || Dataset | state23017 |
| Phenotype | NoPhenotypeAvailable |
| Upregulated in class | na\_neg |
| GeneSet | HALLMARK\_COMPLEMENT |
| Enrichment Score (ES) | -0.48542467 |
| Normalized Enrichment Score (NES) | -1.6376473 |
| Nominal p-value | 0.04045512 |
| FDR q-value | 0.12567222 |
| FWER p-Value | 0.559 |
Table: GSEA Results Summary

  

Fig 1: Enrichment plot: HALLMARK\_COMPLEMENT      
 Profile of the Running ES Score & Positions of GeneSet Members on the Rank Ordered List

  

| PROBE | GENE SYMBOL | GENE\_TITLE | RANK IN GENE LIST | RANK METRIC SCORE | RUNNING ES | CORE ENRICHMENT || 1 | CALM3 |  |  | 82 | 0.308 | -0.0181 | No |
| 2 | ZEB1 |  |  | 84 | 0.303 | 0.0251 | No |
| 3 | CPM |  |  | 289 | -0.263 | -0.0931 | No |
| 4 | PSMB9 |  |  | 341 | -0.267 | -0.0934 | No |
| 5 | S100A13 |  |  | 427 | -0.276 | -0.1184 | No |
| 6 | CASP3 |  |  | 459 | -0.280 | -0.1016 | No |
| 7 | SCG3 |  |  | 488 | -0.283 | -0.0820 | No |
| 8 | CTSL |  |  | 531 | -0.289 | -0.0723 | No |
| 9 | CD59 |  |  | 622 | -0.304 | -0.0972 | No |
| 10 | PDGFB |  |  | 969 | -0.401 | -0.3040 | No |
| 11 | CLU |  |  | 1207 | -0.592 | -0.3997 | Yes |
| 12 | SERPINE1 |  |  | 1218 | -0.617 | -0.3179 | Yes |
| 13 | F3 |  |  | 1241 | -0.673 | -0.2373 | Yes |
| 14 | LGALS3 |  |  | 1287 | -0.878 | -0.1445 | Yes |
| 15 | MT3 |  |  | 1308 | -1.167 | 0.0092 | Yes |
Table: GSEA details [plain text format]

  

Fig 2: HALLMARK\_COMPLEMENT: Random ES distribution      
 Gene set null distribution of ES for **HALLMARK\_COMPLEMENT**
